# Association between poverty and children's working memory abilities in developing countries: a systematic review and meta-analysis

**DOI:** 10.3389/fnut.2023.1067626

**Published:** 2023-05-11

**Authors:** Hari Wahyu Nugroho, Harsono Salimo, Hartono Hartono, Moh. Abdul Hakim, Ari Probandari

**Affiliations:** ^1^Doctoral Program of Public Health, Faculty of Medicine, Universitas Sebelas Maret, Surakarta, Indonesia; ^2^Department of Public Health, Faculty of Medicine, Universitas Sebelas Maret, Surakarta, Indonesia

**Keywords:** poverty, socioeconomic status, working memory, children, developing countries

## Abstract

**Introduction:**

Working memory is critical in cognitive skills, especially among children. Children's ability to count and complete cognitive tasks are strongly associated with working memory abilities. Recent studies indicated that in addition to health factors, socioeconomic status also has a significant impact on children's working memory capacity. Despite these, evidence on the effects of socioeconomic status on working memory from developing countries yielded a somewhat puzzling picture.

**Methods:**

This systematic review and meta-analysis provide a comprehensive summary of the recent evidence concerning the socioeconomic status determinants of children's working memory in developing countries. We searched via Cochrane library, ScienceDirect, Scopus, PubMed, and ProQuest. The initial search terms were [“socioeconomic”, “socio-economic”, “socioeconomic status”, “socio-economic status”, “income”, “poverty”, “disadvantaged”, “disparity”] AND [“working memory”, “short term memory”, “short-term memory”, “cognitive”, “achievement”, “performance”] AND [“child^*^”, “school child^*^”]. Odds ratio (for categorical outcome data) or standardized mean differences (for continuous data) and their 95% confidence intervals were calculated from the data generated.

**Results:**

This meta-analysis included five studies from 4 developing countries with a total of 4,551 subjects. Poverty was associated with a lower working memory score (OR: 3.12; 95% CI: 2.66, 3.65; *p* < 0.001). Another finding from 2 studies in this meta-analysis was that low mother education was associated with a lower score of working memory (OR: 3.26, 95% CI: 2.86, 3.71; *p* < 0.001).

**Discussion:**

Poverty and low level of mothers' education were significant risk factors for lowering working memory among children in developing countries.

**Systematic review registration:**

https://www.crd.york.ac.uk/prospero/, identifier: CRD42021270683.

## 1. Introduction

Less poverty is one of the goals within the framework of the Sustainable Development Goals (SDGs) agenda of 2030. Thus, it is believed that nutrition takes a place at the center of the SDGs. As such, with good nutrition comes less poverty which is in accordance with SGD 1. However, it has been widely recognized that poverty impacts the nutritional and health status (1) and also the cognition of populations (2, 3), particularly for children. Cognitive skills are some of the important indicators of the health status of children (1).

Working memory is type of memory defined as the capability to keep small amount of information in limited short time (4, 5). Working memory plays a critical role in cognitive skills, especially among children (4, 6, 7). Children's ability to count and complete cognitive tasks are strongly associated with working memory capacity (8, 9). Working memory also important in order to develop executive function that contribute to school achievement, such as solve problem effectively, remember number in mathematics, combine words in reading. In addition, working memory plays important rules in cognitive ability (10, 11). The tests can be used to assess working memory are Continuous Performance Test (CPT), Direct and Indirect Digits Test, Wechsler Memory Scale (WMS), Visual Organization Task (VOT), Test of Memory Malingering (TOMM Test Of Variables of Attention (TOVA) (8).

Recent studies indicated that, in addition to health factors, socioeconomic status also has a significant impact on children's working memory capacity (12, 13). For instance, several studies have shown that socioeconomic status influences children's cognitive development, academic achievement, and structural brain development (14, 15). Despite these, evidence on the effects of socioeconomic status on working memory from developing countries yielded a somewhat puzzling picture. Based on a sample of children aged 6–7 years in Brazil, Engel et al. (16), socioeconomic status was suggested to demonstrate no significant effect on working memory (16). Another study result also reported that socioeconomic status did not influence children's working memory (17). On contrary, Aurino et al. (18) reported that socioeconomic status, especially food security, had a relationship with the working memory ability of children aged 7.7 years old on average in Ghana. Another study by Sturge-Apple et al. (19) suggested an indirect effect in which socioeconomic status significantly affected the working memory ability of a 3-year-old child mediated by the mother's working memory. Indeed, several systematic reviews highlight the relationship between socioeconomic status with children's working memory and cognitive ability (13, 14, 20), Regarding specific working memory, Lawson et al. (20) meta-analysis study reported that socioeconomic status had a significant association with children cognition (20). Another meta-analysis, specifically reviewing the association between socioeconomic status and children's working memory, but not specific in developing countries, reported that low socioeconomic status was associated with low working memory in Children (13). Given these inconsistent findings and also needs to asses specific measurement and setting, a systematic review to assess socioeconomic determinants of working memory in children in developing countries is essentially needed by policymakers, researchers, and pediatric practitioners from developing countries.

## 2. Methods

### 2.1. Protocol, registration, and reporting standards

The PROSPERO International Prospective Register of Systematic Review reference has enlisted this protocol with registration number CRD42021270683. We used Preferred Reporting Items for Systematic Review and Meta-Analysis Protocols (PRISMA-P) in this review (21).

### 2.2. Study eligibility criteria

#### 2.2.1. Population, exposure, and outcome

Population, Exposure, and Outcome (PEO) were used to design inclusion criteria in this study. The population was children aged 0–18 years old in developing countries. The exposure was socioeconomic status, and we included any indicators assessment of socioeconomic status. The outcome was working memory performance, defined by any quantified assessment to asses working memory (e.g., Forwards Digit Recall, Backwards Digit Recall, Counting Recall, any “two-back” task, Corsi). Studies with other cognitive or executive function tasks were excluded. This review will address “socioeconomic status,” defined as the conditions of social and financial of the family and also the environment, including the level of education of parents, the income of the family, the total of family members, and environmental wealth. Working memory must be measured quantitatively with a specific test of working memory that was already validated. The term “short-term memory” will also be considered as working memory. The population of interest will include children aged 0–18 years old from economically developing countries. Studies using primary and secondary data from big data will be included. All studies included in the review must be in English and present quantitative data on working memory from observational designs both cohort and cross-sectional studies. Studies designed as a discussion or a review paper will be eliminated, although the reference lists will be screened for suitable primary studies. Inclusion criteria: (a) study measuring working memory, (b) report associations between socioeconomic status and working memory, (c) study sample which was children aged 0–18 years old from developing countries, (d) studies which were published between 2011 and 2021. The study measuring other executive functions, not only working memory, and consisting of children with special needs were excluded.

#### 2.2.2. Study designs

Studies were declared as eligible if they used observational design both cross-sectional and longitudinal or any intervention design. Studies with qualitative data were excluded. Only published studies were eligible.

### 2.3. Search and selection procedures

Guidance from the Cochrane Collaboration (22) was used in this study. We used the electronic database of peer-reviewed journal articles, such as Cochrane Library, ScienceDirect, Scopus, PubMed, and ProQuest. The initial search terms used in this study: [“socioeconomic”, “socio-economic”, “socioeconomic status”, “socio-economic status”, “income”, “poverty”, “disadvantaged”, “disparity”] AND [“working memory”, “short term memory”, “short-term memory”, “cognitive”, “achievement”, “performance”] AND [“child^*^”, “school child”].

#### 2.3.1. Initial screening

The same literature might be determined in several different databases so an EndNote database was applied to reserve retrieved literature and correspondent entries were taken out (23). Preference was presumed to peer-reviewed information and the hierarchy of research study design if in any case, more than one report type was retrieved for the same study; however, further details were withdrawn from the distinct citations where suitable. The titles and abstracts of articles detected through the search methods were primarily screened upon the inclusion criteria for the review to find out all potentially relevant studies. Papers were assigned as either “not relevant” or “potential”. Titles and abstracts contributing to a difference of opinion between reviewers at this point were involved.

#### 2.3.2. Second screening

Full texts of the literature considered potentially relevant, or conducing to a difference of opinion between reviewers, were afterward acquired. A standardized checklist of the eligibility criteria was then accustomed to deciding on inclusion. The second screening was carried out by both the lead reviewer and a second reviewer. In the case of disagreement regarding inclusion, a consensus was reached through discussion amongst the full research team. Sources for excluded studies and the reason for their non-acceptance were reserved in a separate folder of the project EndNote database.

### 2.4. Data extraction

Subsequently, for the validation of literature suitable for inclusion in the review, a bespoke data abstraction framework was applied as a template for recording significant study characteristics. This information included details, as appropriate, on study design, number of participants, socioeconomic measurement, participant demographics, setting, time period, working memory or short-term memory measurement, perspective, analysis, results, and quality appraisal. Data were extracted by the lead reviewer and examined by a second reviewer. The data was tabulated to create a Microsoft Excel spreadsheet summary, thus facilitating accurate comparison between studies. To assure a comprehensive record of relevant, accurate information, the abstraction tool was directed at a small sample of literature selected for inclusion in the review and modified as necessary. Studies similar in terms of population and recorded outcome measures were categorized together in the summary for quality assessment and data synthesis as properly. According to the hierarchy of research study design, discovery is given better preference in descending order from randomized controlled trials, cohort studies, case-control studies, cross-sectional studies, and case reports. In occurrences where inadequate details had been provided to permit complete data extraction or quality appraisal, the study authors were addressed for further information.

### 2.5. Risk of bias in individual studies

Identified studies that met the publication criteria were grouped into cohort studies and case-control studies. These studies were then assessed independently for methodological validity by two reviewers, prior to inclusion in the review using PRISMA.

### 2.6. Quality assessment

Quality assessment (Table 1) was performed by using the CASP critical appraisal (24). Overall, of the eight studies assessed by CASP critical appraisal, one study was rated as having good quality while the other four studies were rated as having fair quality. None of the studies was reported using a reporting checklist to report their studies.

**Table 1:** Quality assessment of observational cohort and cross-sectional studies (NHLBI).

**Question**	**Fernald 2011**	**Lipina2013**	**Da Rosa Picolo2016**	**Prado2017**	**Kolinsky 2020**
1. Did the study address a clearly focused issue?	Y	Y	Y	Y	Y
2. Was the cohort recruited in an acceptable way?	Y	Y	Y	Y	Y
3. Was the exposure accurately Yes measured to minimize bias?	Y	Y	Y	Y	Y
4. Was the outcome accurately measured to minimize bias?	Y	Y	Y	Y	Y
5. Have the authors identified all important confounding factors?	Y	Y	Y	Y	Y
Have they taken account of the confounding factors in the design and/or analysis?	Y	Y	Y	Y	Y
6. Was the follow up of subjects complete enough?	Y	CT	Y	Y	CT
Was the follow up of subjects long enough?	Y	CT	Y	Y	CT
7. What are the results of this study?	R	R	R	R	R
8. How precise are the results?	R	R	R	R	R
9. Do you believe the results?	Y	Y	Y	Y	Y
10. Can the results be applied to the local population?	Y	Y	Y	Y	Y
11. Do the results of this study fit with other available evidence?	Y	Y	Y	Y	CT
12. What are the implications of this study for practice?	Y	Y	Y	CT	Y
Overall quality	Good	Fair	Fair	Fair	Fair

Y, Yes, CT, Cannot Tell; N, No; R, Reported.

### 2.7. Validity and reliability of the review

The first reviewer (a) screened all eligible abstracts and full texts, and the second reviewer (b) screened a random 20% of excluded abstracts and full texts. (a) extracted all data and then (b) checked the data extraction and risk of bias assessments. Each reviewer assessed the risk of bias for 50% of all included studies. Any disagreements were resolved through discussion.

### 2.8. Data synthesis

Odds ratio (for categorical outcome data) or standardized mean differences (for continuous data) and their 95% confidence intervals were calculated from the data generated. Heterogeneity between combined studies was tested using the standard chi-square test. Findings were presented in narrative form.

There are a set of guidelines, incorporating specialized tools and techniques, and a broader framework has been developed by the Economic and Social Research Council, for the configuration of an apparent (clear) reliable narrative synthesis report (25). This framework was applied to design a narrative synthesis of the quantitative data in four main stages:

- Constructing a novel theory of what socioeconomic factors may influence the working memory of the children; how socioeconomic factors may influence the working memory of children.
- Composing an early synthesis of the outcome of included literature.
- Enquiring relationships and any similarity recognized within and between studies.
- Evaluating the extent to which the synthesis of this data can be regarded as robust.

Meta-analysis was conducted to combine and synthesize results data across studies. A fixed model was conducted if the variance was low, but if the variance was moderate, a random effects model was conducted. Heterogeneity was calculated using the χ^2^ test, and if the *p*-value was < 0.10, it would be considered evidence of heterogeneity. The I^2^ statistic was used to assess the percentage of variation. if the I^2^ value was more than 30%, it would be considered moderate heterogeneity, and if the I^2^ value was more than 50%, it would be considered severe. Funnel plots and Egger's test of bias would assess publication bias (26).

## 3. Results

### 3.1. Study selection

**Figure 1:**
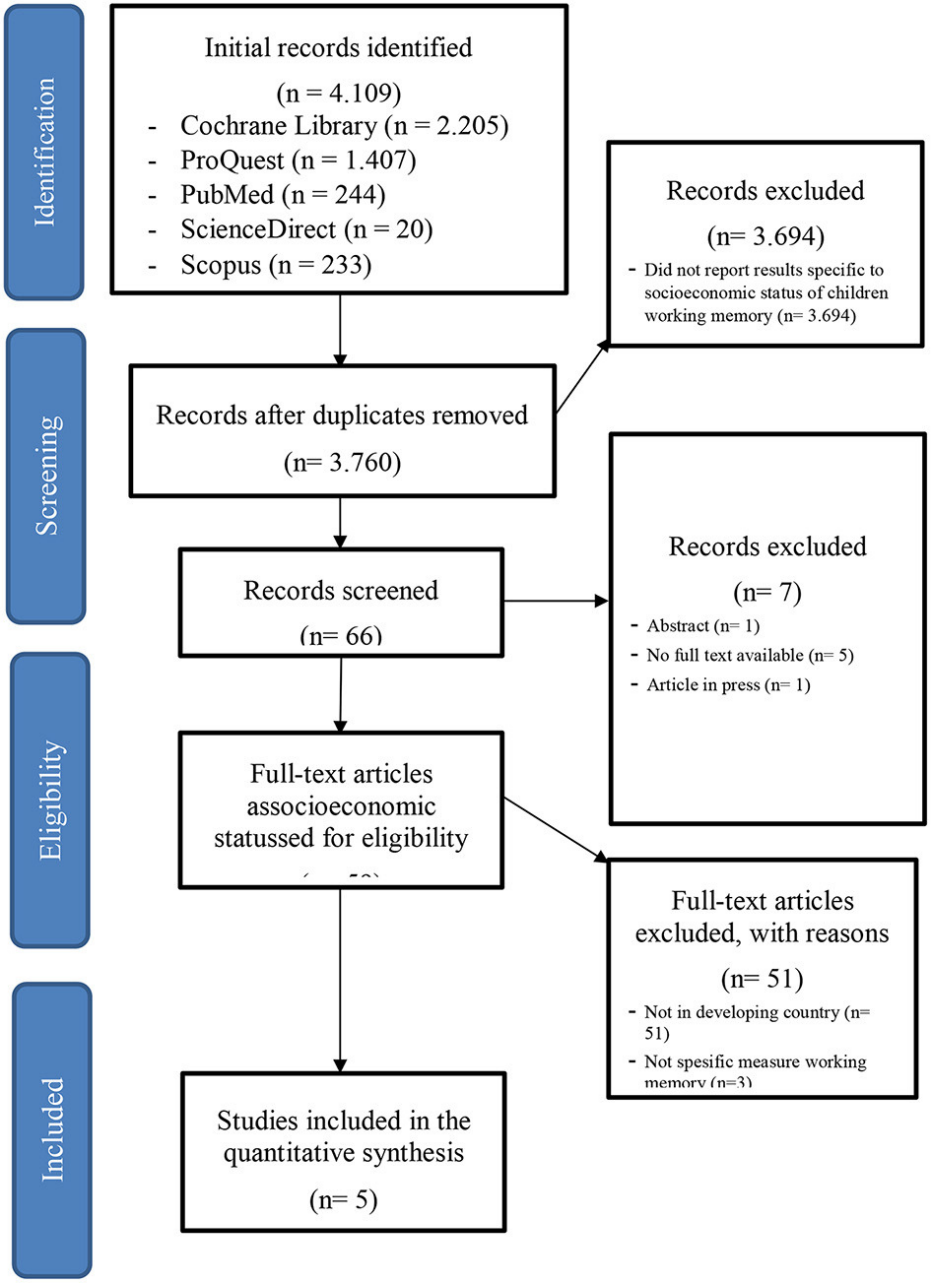
PRISMA 2009 flow diagram for all included studies.

The study selection process was performed based on the PRISMA diagram. A total of five studies, out of 4,109 records, were eligible for the review. Figure 1 shows the selection process for all included studies.

### 3.2. Study characteristics

A total of 5 studies were included with a total sample size of 4,551 (minimum: 70; maximum: 2,879). Three studies were conducted in schools, while the others were in communities. Detailed characteristics of the included studies are provided in Table 2.

**Table 2:** Characteristics of included studies.

**Study No**.	**Study ID**	**Location**	**Study period**	**Setting**	**Working memory assessment**	**Study design**	**Sample size**
1	Fernald 2011	Madagascar	May and July 2007	A nationally representative sample of 3–6-year-old children in 150 communities of Madagascar	Sub-test of the ESB5	Secondary data	1,232
2	Lipina 2013	Argentina	2009	Three school districts of the City of Buenos Aires	Stroop-like Butterfly /Frog and self-ordered search	Cross sectional study	250
3	daRosa Piccolo 2016	Brazil	2009	Low-income community in a city in southern Brazil	Child Brief Neuropsychological Assessment Battery (NEUPSILIN- INF)	Cross sectional study	70
4	Prado 2017	Indonesia	March to April 2019	Prenatal care clinics held by midwives	Digit span forward and backward	Clinical trial	2,879
5	Kolinski 2020	Brazil	Mid- May to the beginning of July	Three schools located in a small town in the South of Brazil (state of Rio Grande doSul)	Serial-order reconstruction task	Cross sectional study	120

### 3.3. Meta-analysis

#### 3.3.1. Summary of effects

**Figure 2:**
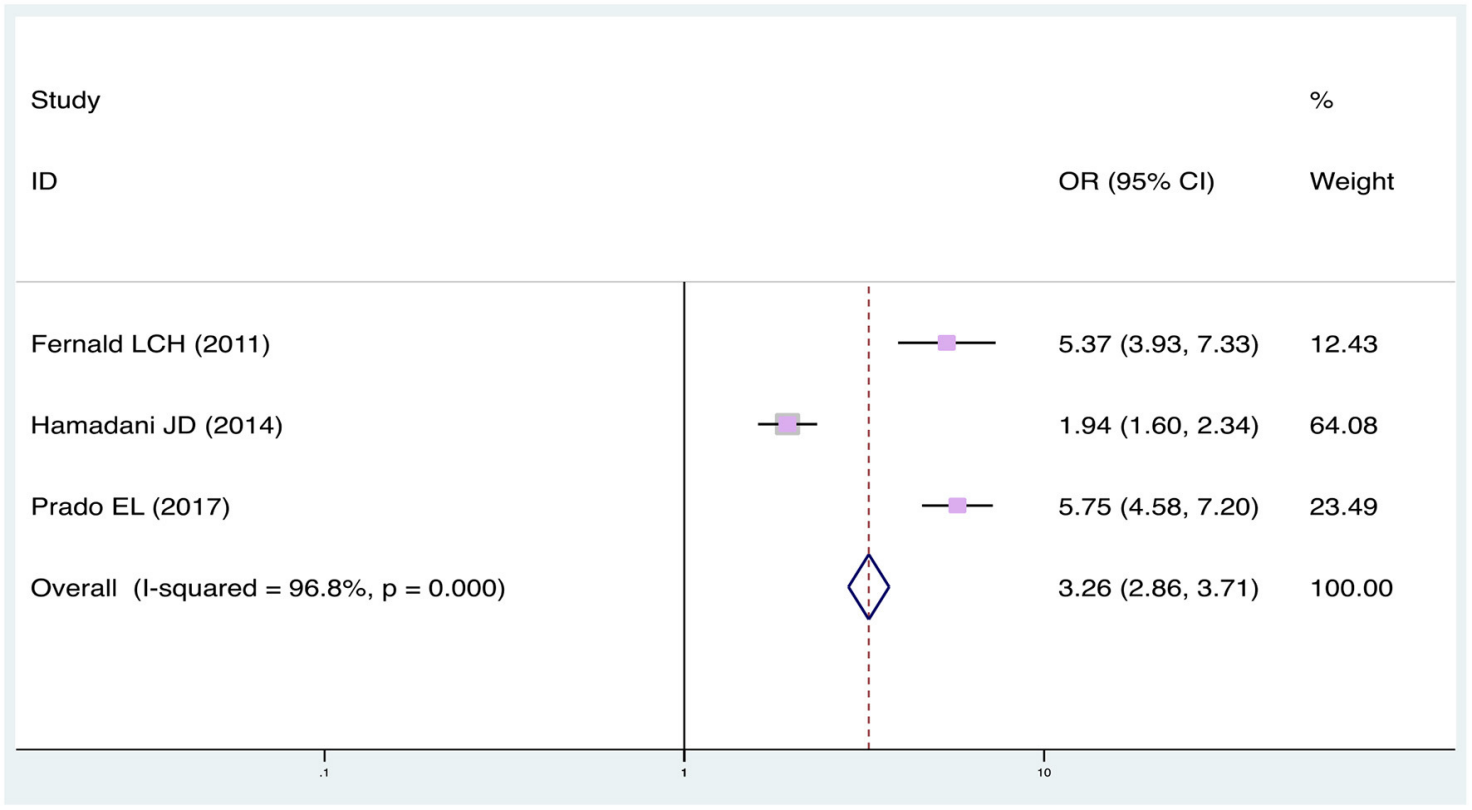
Meta-analysis of the association between mother education and working memory disorder (sorted by effect size). CI, Confidence Interval.

**Figure 3:**
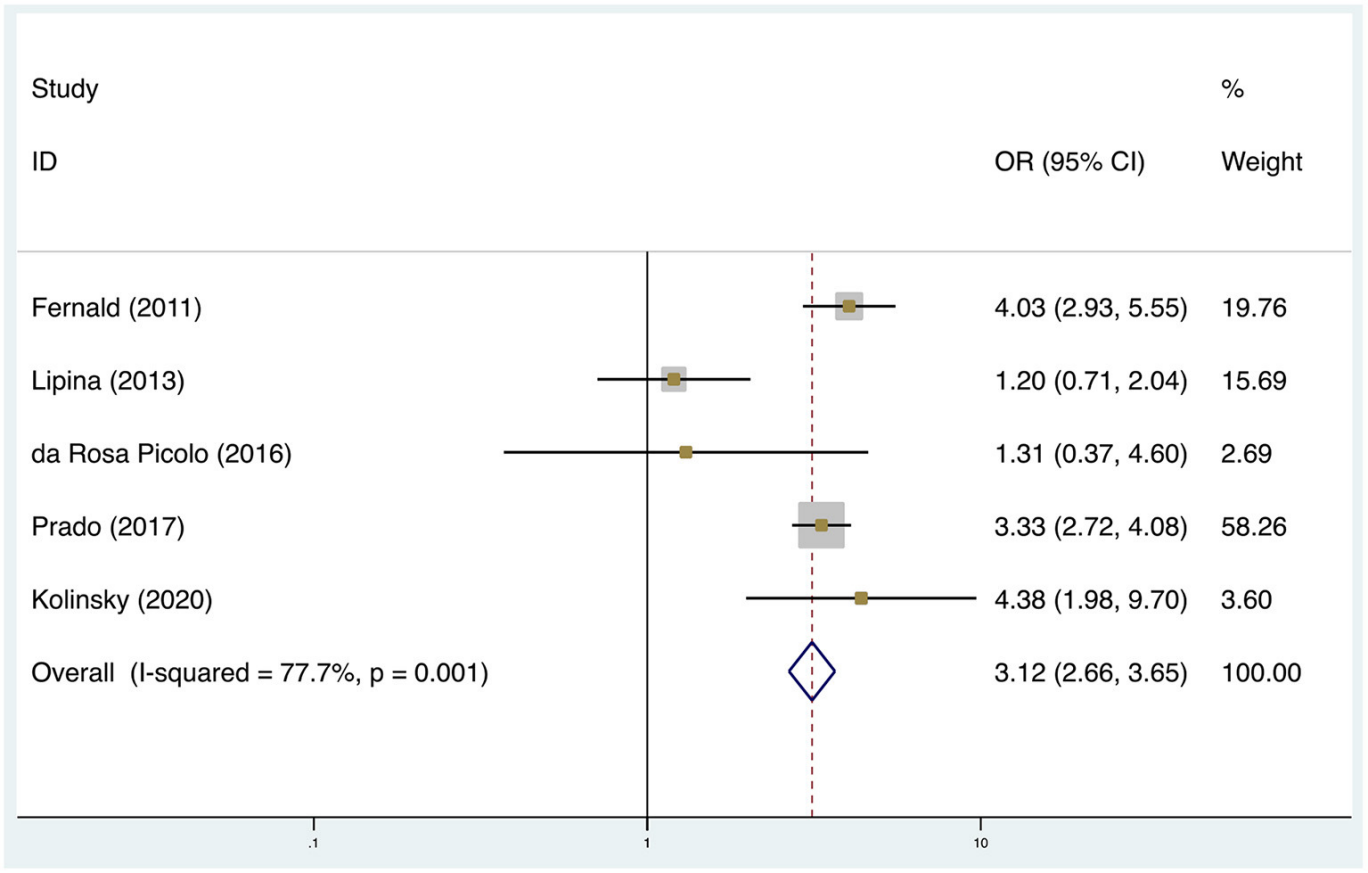
Meta-analysis of the association between socioeconomic status and working memory disorder (sorted by effect size). CI, Confidence Interval.

A meta-analysis was performed for two risk factors (Figures 2, 3), of which, higher mother education (2 studies; OR: 3.26, 95% CI: 2.86, 3.71; *p* < 0.001) and higher socioeconomic status (5 studies; OR: 3.12; 95% CI: 2.66, 3.65; *p* < 0.001) significantly increased the odds of working memory disorder. Factors which increased the likelihood of working memory disorder, but were not significant in the meta-analysis, included age (1 study; OR: 1.41, 95% CI: 0.94, 2.11; *p* < 0.001), father education (1 study; OR: 0.88, 95% CI: 0.66, 1.18; *p* < 0.001), mother depression (2 studies; OR: 0.82, 95% CI: 0.57, 1.18; *p* = 0.007).

#### 3.3.2. Heterogeneity

Heterogeneity was high overall. I^2^ was 77.7% in socioeconomic status and 96.8% in mother education.

#### 3.3.3. Sensitivity analysis

An increase in the pooled effect estimates was observed for socioeconomic status by excluding single studies during sensitivity analysis. The socioeconomic status effect size and 95% CI was 2.21 (1.99 to 2.145).

#### 3.3.4. Publication bias

**Figure 4:**
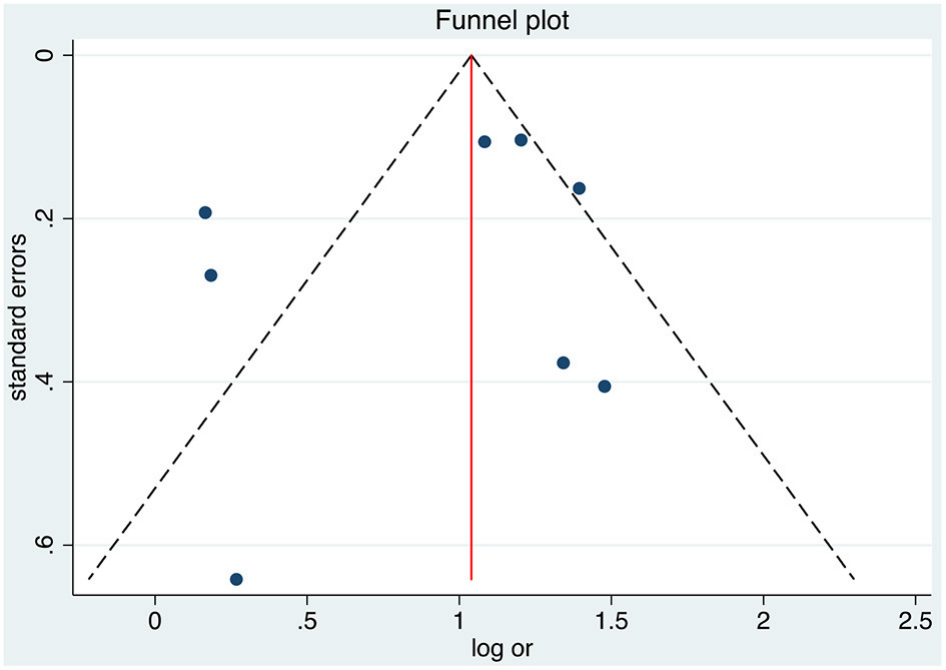
Funnel plot illustrating publication bias associoeconomic statussment for socioeconomic status as risk factor for working memory disorder.

Publication bias was performed for each meta-analysis using a funnel plot, plots of the trials' effect estimated against sample size, which may be useful to assess the validity of meta-analysis, and Egger's tests, which estimated whether the association between study size and effect estimates was greater than expected by chance (26). Publication bias using Egger's regression test did not show statistical significance for association (Figure 4; 5 studies, *p* = 0.442) with working memory disorder.

## 4. Discussions

This meta-analysis was the first meta-analysis that reviewed the association between socioeconomic status with children's working memory in the setting of developing countries. The Total studies and the total subjects that were eligible in our meta-analysis were five studies with total subjects of 4,551 children. Previous meta-analyses were conducted not specifically in developing countries. This meta-analysis study, which was conducted both in developed and developing countries with a huge number of subjects (*n* = 37,737), reported that poverty was associated with children's working memory impairment with a medium effect (13). This result was consistent with another study (9, 12). In contrast, another study showed that there was no association between poverty and working memory in Children (16).

We found in our meta-analysis that poverty had a strong association with the impairment of children's working memory (OR: 3.12; 95% CI: 2.66, 3.65; *p* < 0.001). Since the 5 eligible studies used different kinds of simple and complex tests of working memory, we conclude that poverty lowered not only the capacity to store information but also the ability to process and analyze information. These findings were consistent with other meta-analysis studies (13).

Our meta-analysis also investigated other determinants that are also associated with children's working memory. We found that mother education in three studies had a strong association with children's working memory, overall OR (3.26, 95% CI: 2.86, 3.71; *p* < 0.001). Higher mother education will increase children's working memory ability. This finding was consistent with other studies (12, 27, 28). Age, maternal depression, and father education were also investigated in our meta-analysis, showing no significant correlation with children's working memory. Since all the studies were cross-sectional, we concluded that poverty lowers children's working memory but does not accumulate. These same findings were also reported in another study (13). Early life psychological stress was also proven to have a significant association with working memory, but it was not clear whether the stress was caused by socioeconomic status (29).

Our meta-analysis indicated significant heterogeneity across the studies. This heterogeneity can be caused by clinical and methodological diversity in each study (30). We conclude that the heterogeneity in our study was caused by the differing method used in the working memory tests and also by differing sample sizes in each study.

Further study needs to be conducted using longitudinal and path analysis methods to comprehensively analyze the clear mechanism and association of socioeconomic status with working memory in children, especially in developing countries, since many factors have been proven to pose significant contributions to working memory ability among children such as socioeconomic status, stress, and mother education level. This finding will lay important information for clinical professionals who work in developing countries so that they can give early precise support to children with risk factors of impaired working memory. The factor indicated as moderator factors between the association of socioeconomic status and children's working memory is stress (31) and the lack of enrichment activities provision for children such as toys and books (32).

### 4.1. Strengths and limitations

This study was the first meta-analysis study that reviewed the association of socioeconomic status with children's working memory specifically in developing countries. The decision to choose a specific study setting in this meta-analysis made the result of our study much more specific to become a reference in developing countries. Nevertheless, this also limited our study, since there were only a few studies involving not too many participants of this kind of study in developing countries.

## 5. Conclusion

This is the first meta-analysis specifically in developing countries to review determinant factor of working memory. Consistent with the findings in developed countries, our meta-analysis showed that lower socioeconomic status and low mother education was associated with lower working memory in children from developing countries. These findings are important for all stake holder to support children in developing countries to have better working memory.

## Data availability statement

The raw data supporting the conclusions of this article will be made available by the authors, without undue reservation.

## Author contributions

HN: conceptualization, investigation, and writing—original draft. HS: conceptualization, supervision, and editing. HH and AP: validation and editing. MH: data curation and formal analysis. All authors contributed to the article and approved the submitted version.
